# European recommendations and quality assurance for cytogenomic analysis of haematological neoplasms

**DOI:** 10.1038/s41375-019-0378-z

**Published:** 2019-01-29

**Authors:** K. A. Rack, E. van den Berg, C. Haferlach, H. B. Beverloo, D. Costa, B. Espinet, N. Foot, S. Jeffries, K. Martin, S. O’Connor, J. Schoumans, P. Talley, N. Telford, S. Stioui, Z. Zemanova, R. J. Hastings

**Affiliations:** 1 GenQA, John Radcliffe Hospital, Oxford University Hospitals NHS Trust, Oxford, UK; 2 Department of Genetics University Medical Center Groningen, University of Groningen, Groningen, The Netherlands; 3 MLL-Munich Leukemia Laboratory, Munich, Germany; 4 Department of Clinical Genetics, Erasmus MC, University medical center, Rotterdam, The Netherlands; 5 Hematopathology Section, Hospital Clinic, Barcelona, Spain; 6 Laboratori de Citogenètica Molecular, Servei de Patologia, Grup de Recerca,Translacional en Neoplàsies Hematològiques, Cancer Research Program, imim-Hospital del Mar, Barcelona, Spain; 7 Viapath Genetics laboratories, Guys Hospital, London, UK; 8 West Midlands Regional Genetics Laboratory, Birmingham Women’s Hospital, Birmingham, UK; 9 Department of Cytogenetics, Nottingham University Hospital, Nottingham, UK; 10 Haematological Malignancy Diagnostic Service, St James’s University Hospital, Leeds, UK; 11 Oncogénomique laboratory, Hematology department, Lausanne University Hospital, Vaudois, Switzerland; 12 Oncology Cytogenetics Service, The Christie NHS Foundation Trust, Manchester, UK; 13 Laboratorio di Citogenetica e genetica moleculaire, Laboratorio Analisi, Humanitas Research Hospital, Rozzano, Milan, Italy; 14 Prague Center of Oncocytogenetics, Institute of Clinical Biochemistry and Laboratory Diagnostics, General University Hospital and First Faculty of Medicine, Charles University in Prague, Prague, Czech Republic; 15 GenQA, John Radcliffe Hospital, Oxford University Hospitals NHS Trust, Oxford, UK

**Keywords:** Genetics, Cancer

## Abstract

Cytogenomic investigations of haematological neoplasms, including chromosome banding analysis, fluorescence in situ hybridisation (FISH) and microarray analyses have become increasingly important in the clinical management of patients with haematological neoplasms. The widespread implementation of these techniques in genetic diagnostics has highlighted the need for guidance on the essential criteria to follow when providing cytogenomic testing, regardless of choice of methodology. These recommendations provide an updated, practical and easily available document that will assist laboratories in the choice of testing and methodology enabling them to operate within acceptable standards and maintain a quality service.

## Introduction

Haematological neoplasms are classified according to World Health Organisation (WHO) classification of tumours in myeloid and lymphoid tissues, some of which are defined by the presence of distinct genetic abnormalities [1]. The detection of clonal abnormalities provides support for a neoplastic or premalignant condition and provides important prognostic and therapeutic information [2]. The number of WHO haematological neoplasms defined by genetic abnormalities is steadily increasing as is the number of specific treatment approaches that directly, or indirectly, target genetic abnormalities. Therefore, genetic analysis is an important element in diagnosis, classification, prognostication and monitoring disease response to treatment.

Cytogenomic testing includes chromosome banding analysis, fluorescence in situ hybridisation (FISH) and microarray analyses, and these, together with mutation screening, have become increasingly important in the clinical management of patients. Testing strategies are evolving where array-based techniques and genome wide sequencing strategies are replacing karyotyping and FISH. Currently these techniques are used as standalone tests, or in combination, for evaluation of genetic abnormalities. However new technologies, capable of simultaneously detecting copy number changes, structural variants and mutations, are now available and although these are not currently being used in a diagnostic setting it is expected that this approach will be increasingly used in the future. Recommendations for other molecular testing are outside the scope of this document and are not addressed here. Preferably the results of both cytogenomic and molecular genetics should be integrated to provide an overall comprehensive report stating, if relevant, the cytogenomic prognostic information.

In 2013, specific guidelines for acquired cytogenetics were published, [3] these are updated here to reflect changes in practice. These recommendations were prepared by a panel of 16 experts specialised in cytogenomic testing of haematological neoplasms, all of whom are involved in External Quality Assessments (EQA) for haematological neoplasms. Each section was overseen by a sub-group of the panel. All authors then gave their opinion and the text was adapted in light of the panels’ replies. This process was repeated several times until a consensus emerged. The recommendations were then finalised in a group discussion. Furthermore, opinions were sought from other experts in the field. These recommendations take into account the experience of EQA, good laboratory practice documents, accreditation standards and protocols from different countries, as well as international policy documents. These recommendations apply unless overridden by national/federal laws, regulations and/or standards. In accordance with the terminology recommended by the International System for Human Cytogenomic Nomenclature (ISCN, 2016) [4] cytogenomics is used as a general term in this document. Specific designations are used when needed, otherwise the global term applies. The use of terms such as ‘should’, ‘must’ or ‘essential’ are mandatory requirements (when not in conflict with national law or regulations), while the use of terms such as ‘may’ or ‘could’, are recommended but not mandatory.

These recommendations aim to provide guidance on testing priority and the most appropriate methodologies. This document is organised in two sections: general and specific recommendations. General recommendations address essential criteria to follow when providing cytogenomic testing and specific recommendations advise on the choice of testing and preferred methodology for each entity.

## General recommendations

Essential testing can be achieved by different, or combined, technological approaches. When deciding which method to use, the referral indication plus the advantages and limitations of each technique must be taken into account [5]. It is recognised that laboratories have variable testing strategies depending on available technology and that the testing methodologies covered in these recommendations may be superseded in the future. The methods discussed here comprise the major methods currently used in cytogenomic laboratories.

Service requirements regarding equipment, facilities, staff and diagnostic workload must comply with ISO-15189 [6] standards. Laboratories should provide a robust analytical and interpretive service for neoplastic referrals, have written protocols/standard operating procedures (SOPs), for all aspects of sample processing, based on in-house validated methodologies and/or published guidelines, and participate in an appropriate EQAs. All pre- and post-analytical procedures should also follow written protocols. These guidelines are minimum requirements and professional judgement is of paramount importance. In some circumstances additional analyses/tests should be undertaken to increase confidence in the result. These guidelines should therefore be used in conjunction with relevant clinical trial protocols and/or information from the European LeukemiaNet (ELN) [7].

Referral can be at diagnosis, follow-up prior to or after treatment (including transplantation) and at relapse/transformation. Patients may be in or outside a clinical trial. A close collaboration between genetic laboratories and the referring clinician/haematopathologist is essential to ensure that only clinically relevant samples are analysed and that the most appropriate tests are undertaken. All cytogenomic analysis or checking should involve at least two-independent analysts, at least one of whom has appropriate experience in haematology cytogenomics. In all cases, a suitably qualified person (preferably with professional registration) must confirm that appropriate investigations have been carried out at an acceptable level of quality with respect to the referral reason, and authorise the case report.

### Sample type and processing

#### Choice of sample

For myeloid neoplasms, acute leukaemia and myeloma the tissue of choice for analysis is bone marrow (BM). BM is essential for chromosome analysis, it is only appropriate to use peripheral blood (PB) if there is a significant level of circulating disease [8]. It is advisable to liaise with the referring clinician before reporting a normal analysis for this sample type. For lymphoproliferative disorders such as CLL the preferred tissue is PB, but BM is an alternative if infiltrated. For FISH analysis, BM or PB smears are an alternative.

For the majority of lymphomas the most suitable tissue is lymph node or a relevant biopsy from the primary site. Analysis of BM or PB analysis is not appropriate unless there is morphological/immunophenotypic evidence of infiltration.

#### Sample collection

BM and PB samples are generally taken directly into anti-coagulant tubes, which must be heparin for cell culture and EDTA for DNA–based analysis. Alternatively, for chromosome banding analysis, genetic laboratories can provide clinicians with appropriate transport medium containing an anti-coagulant as this may reduce the risk of sample failure. A sufficient quantity of BM (0.5–1 ml minimum) should be received by the diagnostic laboratory, preferably within 24 h after aspiration. Ideally the first draw should be provided to avoid haemo-dilution of the sample. Lymph nodes and other tissues should be supplied in a sterile container with transport medium. A sufficient quantity of material (5 mm^3^ minimum) should be received by the diagnostic laboratory, preferably the same day.

#### Sample processing

##### Cell culture

Cells should ideally be cultured the day of reception. If it is unclear whether chromosome band analysis is required fixed cultured cells can be stored pending further decisions. Culture choice is dependent on the referral reason and a range of cell culture techniques must be available as several factors, such as addition of mitogens or growth factors and duration of culture, can impact the rate of detection of an abnormal clone. Therefore at least two different cultures should be set-up where possible. When introducing new cultures, laboratories should carry out appropriate assessments of mitotic indices and abnormality rates. With the exception of samples that pose a high risk of infection to laboratory staff, a method for cell counting should be used to establish an optimum culture density of 1–2 × 10^6^ cells/ml. Detailed protocols can be found in numerous text books and manuals [9].

One and/or 2 day cultures are standard for all myeloid disorders. For Myelodysplastic syndromes (MDS) use of specific growth factors or conditioned media may improve quality [10] and cultures with prolonged colcemid exposure can increase the success rate especially in cases with a low cell count [11]. For Myeloproliferative neoplasms (MPN), where BM is not available due to fibrotic marrow, cells washed from a trephine biopsy can produce a result. It may be useful to culture both BM and PB for acute leukaemia (AL) samples as the chromosome resolution of the blasts may vary. A 48h culture should be considered for cases where there is suspicion of a t(8;21) or t(15;17) since, in the experience of many investigators, the aberrant clone is less reliably detected in the 24h culture.

In vitro cell death can be a significant problem for acute lymphoblastic leukaemia/lymphoma (ALL) samples and multiple cultures are recommended where practical. Cell cycle synchronization techniques as well as high colcemid concentrations or prolonged exposure may have a negative impact on metaphase yield due to cell poisoning. At reception, laboratories should consider harvesting one culture and prepare smears or cell suspensions for interphase FISH. Additional cultures include overnight and 2-day cultures, also cultures with addition of growth factor supplements may be useful [12].

For chronic lymphocytic leukaemia (CLL) analysis of PB is more successful than BM. The spontaneous proliferation rate of CLL cells in vitro is low and in order to maximise the yield of metaphases a 3–5 day culture supplemented with an oligonucleotide and the cytokine IL2 should be performed [13–16]. A 3–5 day culture with phorbol 12-myristate 13-acetate (TPA) may also be useful.

For lymph node specimens 24-h culture is recommended, additional culture with the addition of mitogens may also be considered such as 72-h with TPA or addition of an oligonucleotide and IL2. For other tissues spontaneous mitoses may be difficult to obtain and addition of mitogens or growth factors to cultures as described for lymph nodes is required.

##### Cell selection procedures

For multiple myeloma (MM), due to the low levels of plasma cells in the BM of some patients, genetic analysis should be performed on enriched CD138+ cells or DNA extracted from this cell fraction. PB should only be considered for plasma cell leukaemia. Recommended methods of separation include magnetic bead or flow cytometry but selection can be performed by any suitable validated technique. The quality of the plasma cell selection step should be assessed before hybridisation.

### Chromosome banding analysis

Chromosome banding analysis is mandatory for several disease entities (Table 1). Its main advantage is that it provides whole-genome analysis detecting both numerical and structural abnormalities and permits the identification of clonal evolution and the presence of multiple independent clones. However, the requirement for metaphases means that it is not applicable for all disease entities and the poor quality of metaphase in some cases means that abnormalities may be incorrectly interpreted or remain unknown. Furthermore some abnormalities are cryptic by chromosome banding and require complementary testing to determine their presence.

**Table 1:** Recommended testing for different haematological neoplasms

Disease	Test	Requirement	Suggested methodology	Guidelines
CML	Karyotype	Mandatory	Chromosome banding	Baccarani et al. 2013 [24], 2015 [25]
	*BCR-ABL1* gene fusion	Mandatory	FISH or molecular methods	
	*ABL1* mutation when resistance to therapy	Mandatory	Molecular methods	
MPN	*JAK2, CALR, MPL* mutations depending on referral reason	Indicated	Molecular methods	Gong et al. 2013 [32] Xia and Hassejian 2016 [33]
	Karyotype	Optional	Chromosome banding	WHO 2017 [1]
Myeloid/lymphoid neoplasms with eosinophilia	Recurrent gene fusions involving *PDGFRA, PDGFRB, FGFR1, PCM1-JAK2*	Strongly recommended for most patients	FISH or molecular methods	Butt et al. 2017 [40]
	Karyotype	Recommended in absence of recurrent gene fusion	Chromosome banding	
MDS	Karyotype	Mandatory	Chromosome banding	Malcovati et al. 2013 [41]
	Targeted chromosome abnormalities -5/5q-,-7/7q-, *MECOM* (extended panel + 8,20q-del*TP53*)	Recommended^b^	FISH/ SNP array/ Molecular methods	
	High resolution chromosome analysis and aCN-LOH^c^	Recommended	SNP array	
	Mutation analysis of candidate genes	Recommended	Molecular methods	
AML	Karyotype	Mandatory	Chromosome banding	Döhner et al. 2017 [47]
	Gene mutations:*NMP1,CEBPA, RUNX1, FLT3,TP53, ASXL1*	Mandatory	Molecular methods	
	Recurrent gene fusions: *PML-RARA*, *CBFB-MYH11, RUNX1-RUNX1T*. Gene rearrangements of *KMT2A* and *MECOM*.	Recommended^a^	FISH or molecular methods	
ALL	Recurrent gene fusions (Age-related priority see Table 3)	Mandatory	FISH or molecular methods	Harrison et al. 2010 [57]
	Hyperdiploidy	Recommended	Chromosome banding or SNP-Array/FISH	Moorman et al. 2010 [59]
	Recurrent microdeletions	Recommended in paediatric	MLPA, Array, molecular methods	Harrison et al. 2010 [57]
	Karyotype^d^	Mandatory		Hoelzer et al. 2016 [60]
CLL	Deletion 13q14, *ATM, TP53*, trisomy12	Mandatory	FISH, SNP-array or molecular methods	Hallek et al. 2018 [71]
	*TP53* mutation/IGHV mutational status	Mandatory	Molecular methods	Malcikova et al. 2018 [75], Rosenquist et al. 2017 [76]
	Karyotype	Desirable for clinical trials		Hallek et al. 2018 [71]
Multiple myeloma	t(4;14)^e^, t(14;16), deletion *TP53* ^e^ gain 1q/del(1p)	Recommended	FISH for gene rearrangements	Sonneveld et al. 2016 [82]
	t(11;14), t(14;20), ploidy status (extended panel)		FISH or Array, MLPA for copy number gains and losses	Caers et al. 2018 [83]
Other mature B-cell neoplasms	Recurrent gene rearrangements depending on differential diagnosis		FISH	WHO 2017 [1]
	*MYC* rearrangements for prognostic testing^f^		FISH	

^a^For prognostic impact

^b^In cases of karyotype failure or where morphological suspicion of specific abnormality

^c^aCN-LOH: acquired copy neutral loss of heterozygosity

^d^May not be required for all paediatric B-ALL where only basic risk stratification is required

^e^Minimum testing required

^f^If *MYC* rearrangement is detected *BCL2* and *BCL6* should be undertaken for differential diagnosis between Burkitt lymphoma and a double-hit lymphoma

Expertise in G-banding is assumed throughout this document, but R- and Q-banding may also be used. It is essential that the type of banding used is sufficient for the identification of cytogenetically visible recurrent translocations. Throughout the document, the word ‘score’ is used with the specific meaning of checking for the presence or absence of particular structural or numerical abnormality in a given number of cells.

As the quality of chromosome morphology and resolution of neoplastic metaphases is frequently poor, particularly in leukaemia, and requesting repeat samples is often not an option, no minimum banding quality can be recommended. Laboratories should be capable of analysing cells with different resolutions of chromosome banding. As normal cells with better chromosome morphology may be present, it is important to analyse cells of varying quality in order to maximise the likelihood of detecting a neoplastic clone. Where clarification of chromosome abnormalities is required additional testing, such as FISH or microarray analysis, may be needed.

The ISCN definition of clonality stipulates that an identical structural abnormality or chromosome gain should be present in at least two metaphases while loss of a single chromosome should be identified in at least three metaphases. For chromosome loss care must be taken to exclude cells with artefactual random losses in this score. The finding of a single abnormal metaphase necessitates further screening or testing by another technique to determine clonality, particularly a single cell with trisomy 8 or monosomy chromosome 7 in myeloid neoplasms.

Polyploid and hypodiploid/apparently broken metaphases should not be excluded from the analysis, although cells with loss of >6 chromosomes cannot be considered to be fully analysed unless the loss is part of the clonal change. Laboratories should be aware that co-existing clones and clonal evolution may be present and additional analysis should be undertaken if suspected. Analysis from more than one culture regimen should be considered if no abnormal clone is detected, particularly where the lineage of the neoplastic cells is uncertain.

### Analysis at diagnosis

When no abnormality is found in a diagnostic sample a minimum of 20 metaphases must be examined. This will exclude the presence of a chromosomally abnormal clone of 14% with 95% confidence [17]. Ten metaphases should be fully analysed, with a further ten analysed or counted and scored for relevant structurally abnormal chromosomes. If a normal result is based on examination of fewer than 20 cells, the report must be suitably qualified stating that the analysis cannot reliably exclude a significant clonal abnormality.

When a clonal abnormality is found at diagnosis a minimum of ten metaphases must be analysed, where possible. Where a constitutional chromosome abnormality is suspected, screening additional metaphases may permit the detection of a normal cell line. If a constitutional origin cannot be excluded, analysis of a phytohemagglutinin (PHA) stimulated PB sample may be requested. Consideration should be given to the wider implications for the patient and their family members.

### Follow-up after treatment or in remission

Where cytogenetic follow-up is required, the following strategies are recommended:

- Normal result obtained at diagnosis: further analysis is usually not appropriate.
- Abnormal result obtained at diagnosis: chromosome analysis is appropriate. Normal result obtained: Minimum of 20 metaphases should be analysed or scored for the diagnostic abnormality. Abnormal clone detected: Minimum analysis can be limited to 10 metaphases.
- Where cells were only scored, the report should qualify that the analysis only excludes the presence of that specific abnormality.
- Where possible, for follow-up studies/detection of minimal residual disease, quantitative polymerase chain reaction (RQ-PCR), other molecular technologies as well as multi-parametric flow cytometry are preferable.
- For post-transplantation samples molecular technologies are preferable. FISH can be used if a suitable marker was identified at diagnosis.

### For samples at possible relapse or transformation or secondary haematological neoplasm

If clinical information is suggestive of relapse, refractory disease or a second haematological neoplasm, repeat analyses are indicated and samples should be processed as described for diagnostic samples.

### Interphase and metaphase fluorescent in situ hybridisation (FISH)

Interphase FISH, with an appropriate panel of probes, may be used as a sole or first line test for some neoplasms (Table 1). FISH analysis is useful for neoplasms where metaphases are difficult to obtain and where rapid diagnostic testing is required for therapeutic decision making. FISH is the method of choice where the abnormality is not amenable to other molecular methods due to variable breakpoints (for example IGH), where rearrangement of specific genes involves multiple possible partner genes (for example *KMT2A*), where the abnormality is sub-microscopic (microdeletions) or cryptic by chromosome banding analysis. For deletions laboratories should consider the size range of the critical deleted region to ensure that the FISH probe is capable of reliable detection. Very small copy number changes should be tested by array or other molecular methods. FISH analysis is a useful complementary technique, particularly in combination with array data (Table 1) for the detection of balanced rearrangements, and to clarify chromosome banding analysis. Locus-specific FISH should be considered to confirm or exclude chromosome rearrangements with prognostic implications if their presence is in doubt. The disadvantage of FISH is that it is a targeted analysis and therefore needs to be combined with other testing to provide comprehensive information of chromosome abnormalities.

Any FISH system used in a diagnostic setting must be validated. Thresholds and confidence limits should be established for all FISH probes and probe sets. Validation should include documentation of aberrant signal patterns for a number of normal and abnormal samples to establish the false positive/negative ranges. It is important to establish the thresholds/confidence limits using a variety of different preparations which reflect the typical diagnostic samples (e.g. fixed cells, smears, cytospins, paraffin embedded sections, touch preparations etc.). When applicable, adequate control probes should be included in the hybridisation. Laboratories must be aware of the different types of FISH probes and have documentation available that explains normal and abnormal signal patterns e.g. dual colour break apart probes and dual colour dual fusion probes. There are many publications and text books which provide detailed methodology, expected signal patterns and examples of applications [18].

A minimum analysis of 100 interphase nuclei is recommended for diagnostic samples. For follow-up of previously detected clonal anomalies at least 200 interphase nuclei must be analysed. For minimal residual disease with recurrent rearrangements, RQ-PCR or other molecular technologies are more appropriate. It is recognised that an adequate-positive result can often be obtained from a smaller number of cells. An equivocal finding (i.e. the possibility of a low-level clone close to the threshold cutoff) may need more cells and/or a repeat investigation. Care should be taken when reporting these equivocal findings to avoid over interpretation. When results are just above the cutoff value, the report should state that the clinical significance is unclear. It can be useful to examine metaphases, if present, as analysis of normal metaphases confirms cytogenetic location of the probes used and abnormal metaphases can be invaluable in interpreting unusual signal patterns.

The use of FISH on formalin fixed paraffin embedded (FFPE) sections or touch preparations/smears is an appropriate approach for investigation of specific chromosomal aberrations and has the advantage that tumour tissue can be directly screened [19–21]. When analysing FFPE sections it is important to analyse areas of the tissue containing the tumour cells and not to limit the analysis to one area of the section. Since lymphoma often has a diffuse pattern of infiltration, identification of specific areas of tissue containing the tumour cells prior to analysis is not always required, unless the lymphoma is in situ/focal or there is necrosis present. It should be noted that a high number of T cells and other normal/reactive cells may be present.

The number of cells for analysis of FFPE specimens will vary on a case by case basis; the focus should be on determining the presence or absence of a rearrangement not on specific numbers of cells analysed. Although it is optional to include the number of cells analysed in the report, if reported, it should specifically represent the number of tumour cells analysed. At analysis, care should be taken to note any areas of differential digestion, as tumour cells can be over- or under-digested compared to normal cells, and optimum quality of hybridisation should always be sought. If sub-optimum hybridisation has occurred, repeat deparaffination/FISH is recommended with additional optimisation of the pre-treatment prior to FISH. A common limitation of FFPE FISH is that there may be a large proportion of cells displaying one (or no) signal, consistent with signal dropout (truncation of cell nucleus), and should not be reported. Due to variable signal dropout, and variation in the size of cells, analysis for specific deletions using FFPE tissue needs to be carefully considered and appropriate probe design with controls is essential. It is important not to over-interpret apparent signal loss as actual deletion of the probe or the presence of an unbalanced translocation.

### Genomic arrays

To overcome some of the limitations of chromosome banding analysis and targeted FISH, genome wide screening for the detection of copy number abnormalities (CNA) and acquired copy neutral loss of heterozygosity (aCN-LOH) using microarrays was introduced. This technology can be used as a complementary test or, depending on the neoplasia investigated, as a standalone test. It can be used at diagnosis and follow-up but is not suitable for minimal (measurable) residual disease (MRD) monitoring after treatment (including transplantation).

As no cell culture is required and clone selection due to cell proliferation is avoided it is a valuable technique for pathologies where chromosome banding studies are difficult or impossible to perform, where there are no or insufficient metaphases, where a normal karyotype and normal FISH results are obtained at diagnosis and where chromosome resolution is poor. Arrays are particularly useful for neoplasms where multiple CNAs are tested, replacing multiple FISH analyses, and is increasingly important for the detection of very small CNAs. However, it is important to note that one of the limitations of microarrays is that they cannot exclude some recurrent translocations and so additional testing is required for some diseases.

Microarray analysis allows accurate detection of a number of very small CNAs of prognostic significance and can identifiy genomic instability, including complex genomic aberrations such as chromothripsis. Microarrays containing single nucleotide polymorphisms (SNPs) enables ploidy level to be established and allows the detection of aCN-LOH. These regions often mask point mutations in tumour suppressor genes and therefore serve as important indicators for further molecular investigations such as Next Generation Sequencing (NGS).

It is essential that the sensitivity and resolution of genomic regions harbouring clinically relevant genes are established by a thorough internal validation of known abnormal samples prior to diagnostic use. Specific guidelines for genomic array analysis in acquired haematological neoplastic disorders were published in 2016 [22].

### Testing strategies

Each haematological neoplasm is defined by a spectrum of genetic aberrations of diagnostic and/or prognostic significance and the testing required is pathology specific. Essential testing is discussed within the specific disease sections and summarised in Table 1. Recommended testing can be achieved by different (or combined) technological approaches (Table 2) and analytical strategies will vary depending on local policy and infrastructure. It is recognised that testing is a rapidly evolving field and that the introduction of new technologies will vary between laboratories and from country to country. There are now methods which can simultaneously detect numerical and structural abnormalities as well as mutations in one assay [23]. While this approach has not yet been introduced extensively into routine testing, it is acknowledged that this may change in the future and therefore this has been included as an alternative strategy in Table 2.

**Table 2:** Alternative testing strategies

Testing requirement	Alternative strategies for testing
Whole-genome numerical and structural abnormalities	Chromosome banding plus FISH/molecular testing for recurrent cryptic structural abnormalities or Array/NGS copy number analysis plus FISH/molecular analysis for recurrent structural abnormalities or Whole-genome NGS analysis including copy number and structural abnormalities
Targeted region-specific analysis for recurrent structural abnormalities deletions, gain, translocations	FISH for copy number and structural abnormalities or FISH for copy number plus molecular techniques for structural abnormalities or Molecular-based copy number (e.g. MLPA, PCR) plus FISH/molecular analysis for recurrent structural abnormalities or Targeted Array/NGS copy number analysis plus FISH/molecular analysis for structural abnormalities or Targeted NGS analysis, including copy number and structural abnormalities

*NGS* next generation sequencing

## Disease-specific recommendations

In this section we provide the recommended disease-specific cytogenomic testing required. This section includes:

- Chronic myeloid leukaemia (CML) at diagnosis and follow-up for staging purposes or to monitor therapy efficacy.
- Chronic myeloproliferative neoplasms (MPN) for selected cases or where there is acute leukaemic transformation.
- Myeloid/lymphoid neoplasms with eosinophilia at diagnosis.
- Myelodysplastic syndrome (MDS) at diagnosis, at disease progression and after treatment.
- MDS/MPN and germ line predisposition.
- Acute leukaemia at diagnosis and follow-up.
- Chronic lymphocytic leukaemia (CLL) for prognostication at time of treatment and/or clinical progression or to aid in differential diagnosis.
- Multiple myeloma for prognostication.
- Lymphoma and other lymphoproliferative disorders (LPD) in selected cases at diagnosis, follow-up or relapse and to aid in differential diagnosis or prognostication.

In a proportion of cases the diagnosis is not known at referral. Some testing, such as chromosome banding analysis and any rapid testing requested, needs to be initiated at sample reception and thus, in the absence of any further clinical information, sufficient testing should be undertaken taking into account the different diagnoses possible. Fixed cell cultures can be stored for analysis pending further information. Other testing such as non-urgent FISH or molecular testing can be initiated once more information is available.

### Chronic myeloid leukaemia (CML)

The t(9;22)(q34;q11) is the hallmark of CML and results in the generation of the Philadelphia chromosome (Ph), der(22)t(9;22)(q34;q11). This translocation is detected in 90–95% of CML cases at diagnosis. The remaining 5–10% have a variant t(9;22) involving additional chromosomes or have a cryptic rearrangement, usually an insertion, resulting in the presence of a *BCR-ABL1* gene fusion.

In some patients additional chromosome abnormalities (ACA) in Ph positive (Ph+) cells are present at diagnosis. It is important to detect these as they may identify patients at risk of disease acceleration [24–27] and aid the interpretation of results from subsequent samples. ACA can be defined as ‘major route’ abnormalities or ‘minor route’ abnormalities [27, 28]. Abnormalities in Ph+ cells, arising during the course of the disease (clonal evolution), are an independent poor prognostic factor for survival in both chronic and accelerated phases (AP) of CML [29, 30]. WHO 2017 classification defines new criteria for AP that includes the presence of specific ACA [1]. *Major route* abnormalities (a second Ph chromosome, trisomy 8, isochromosome 17q, trisomy 19), complex karyotype and abnormalities of 3q26.2 are each criterion for accelerated phase as is the appearance of any new clonal chromosomal abnormality in Ph+ cells that occurs during therapy.

The ELN recommendations for analysis of CML should be followed [24, 25]. Chromosome analysis is mandatory for CML at diagnosis. It is strongly recommended that 20 cells are fully analysed to exclude the presence of ACA. If a variant translocation is detected then *BCR*-*ABL1* fusion must be confirmed by FISH or reverse transcription PCR (RT-PCR). A rapid preliminary test may be undertaken with *BCR/ABL1* FISH probe using a direct harvest or smears. It is recommended that dual fusion probes are used as they give a more reliable informative signal pattern than the ES (extra signal) probe. FISH is mandatory for cases with an insufficient number of metaphases and for cases with a normal karyotype, to exclude a cryptic abnormality. If no *BCR-ABL1* fusion can be detected by FISH, and CML is still clinically suspected, then RT-PCR and/or molecular testing for mutations associated with other myeloproliferative neoplasms (MPN) is required (see MPN section below).

Response to treatment can be determined cytogenetically by monitoring the reduction in the number of Ph+ cells. Follow-up chromosome analysis should be performed at 3, 6 and 12 months post treatment until a complete cytogenetic response (CCyR) has been achieved and at least 20 cells must be fully analysed for disease monitoring [25]. More frequent monitoring may be required for patients in whom additional abnormalities were found at diagnosis as these may have an adverse response to TKI therapy [24]. If a cryptic *BCR-ABL1* rearrangement was detected at diagnosis then follow-up must be done by FISH or molecular methods. Once CCyR has been achieved chromosome analysis can be replaced by FISH. Accurate interpretation of FISH follow-up requires prior knowledge of the signal pattern at diagnosis, and cases with only a single fusion signal cannot reliably be monitored by FISH [25]. If RQ-PCR methodology is standardised, and expressed according to the International Scale, then response can be assessed using only RQ-PCR [25]. For follow-up, peripheral blood samples are more appropriate for the subsequent study of response to treatment.

Although the purpose of genetic analysis after therapy is to monitor the level of Ph+ cells, it is also recognised that new clonal abnormalities are occasionally detected in Ph− cells and these should be followed up in subsequent samples. Whilst unexpected additional abnormalities may be transient and of no clinical significance [24, 31], a minority (2–10%) of these CML patients go on to develop clinically evident MDS/AML, a risk associated particularly with Ph− clones harbouring deletions of 7q or monosomy 7 [24].

### Myeloproliferative neoplasms (MPN)

MPN is a heterogeneous group of disorders characterised by proliferation of one or more myeloid lineages. The WHO classification of MPN includes CML (covered in the previous section), chronic neutrophilic leukaemia (CNL), polycythaemia vera (PV), primary myelofibrosis (PMF), essential thrombocythaemia (ET), chronic eosinophilic leukaemia NOS and mastocytosis.

For many referrals of MPN, the differential diagnosis is CML and the important genetic requirement is exclusion of the *BCR*-*ABL1* rearrangement. Mutation analysis of *JAK2, CALR* and *MPL* is particularly important in the diagnostic workup of some MPN [1, 32, 33]. Currently these mutations are considered diagnostic, but may, in time, influence treatment decisions and prognosis. Cytogenetic studies are not essential and many laboratories do not offer chromosome analysis because of this lack of specificity. However, recently the karyotype has been included in the DIPSSPlus prognostic scoring systems in primary PMF [34–38] and it has been suggested that it may also be a useful indicator in PMF post PV or ET [39].

### Myeloid/lymphoid neoplasms with eosinophilia and abnormalities of *PDGFRA, PDGFRB* and *FGFR1* or with *PCM1-JAK2*

A distinct classification of myeloid and lymphoid neoplasms associated with eosinophilia and rearrangements of *PDGFRA, PDGFRB*, *FGFR1* or with a *PCM1*-*JAK2* rearrangement is described in the WHO 2017 [1]. This disorder is associated with recurrent rearrangements which have variable responsiveness to targeted drugs. *FIP1L1-PDGFRA*, *PCM1*-*JAK2* gene fusions and *PDGFRB* and *FGFR1* and *PCM1*-*JAK2* gene rearrangements should be excluded by FISH or RT-PCR [40]. Since almost all tyrosine kinase gene fusions, apart from *FIP1L1*-*PDGFRA*, are associated with visible chromosome rearrangements chromosome banding studies may be performed for screening.

### Myelodysplastic syndromes (MDS)

Cytogenetic analysis of bone marrow aspirate should be performed in all patients with suspected MDS for whom bone marrow examination is indicated [41]. Selected recurrent abnormalities are recognised as presumptive evidence of MDS, even in the absence of definitive morphologic features [1]. The cytogenetic risk group is included in the IPSS-R prognostic scoring system [42, 43]. Although this is based on chromosome analysis, abnormalities detected by FISH or array may provide prognostic information.

SNP-array is emerging as an important tool in MDS. aCN-LOH as a sole genomic abnormality is a recurrent finding in MDS with normal karyotypes and these regions often harbour point mutations in genes associated with poor outcome in MDS such as *ASXL1*, *EZH2*, *TP53* and *RUNX1*. Mutation screening with NGS panels is therefore strongly recommended in cases with aCN-LOH and a good IPSS-R score.

If no, or insufficient, metaphases are obtained, array or FISH analysis for monosomy 5/deletion of 5q and monosomy 7/deletion 7q, must be undertaken. Analysis can be extended to include trisomy 8, *TP53* deletion and 20q deletion [41]. Where morphology or immunophenotype suggests a deletion of 5q or *MECOM* gene rearrangement (poor risk according to IPSS-R), in the absence of cytogenetic abnormality, further testing must be undertaken. Point mutations in the *TP53* gene have been reported to cause resistance to lenalidomide in 5q deletion MDS patients. Therefore mutation analysis of *TP53* is indicated in patients with low or absent treatment response, or should be recommended in the report if not performed in the laboratory.

Patients with aplastic anaemia (AA) may be referred to rule out MDS. AA results from bone marrow failure and is not a neoplastic disorder. Consideration should be given to the possibility of Fanconi anaemia, especially in childhood aplastic anaemia and liaison with the referring clinician with regard to appropriate testing is important. SNP array can be used as a complementary test to detect recurrent abnormalities observed in AA that will not be detected by chromosome banding analysis, such as aCN-LOH for 6p. aCN-LOH 6p is not a clonal abnormality related to neoplasia but is thought to arise as an escape mechanism in this autoimmune disorder [44].

### Myelodysplastic/myeloproliferative neoplasms

This category includes myeloid neoplasms with overlapping features of MDS and MPN and encompasses chronic myelomonocytic leukaemia (CMML), atypical CML (aCML), juvenile myelomonocytic leukaemia (JMML), and MDS/MPN with ring sideroblasts and thrombocytosis (MDS/MPN-RS-T) [1]. Some of these diseases are characterized by mutations in certain genes like *SF3B1* in MDS/MPN-RS-T. Chromosome banding analysis of referrals within the MDS/MPN classification group can be considered within the same category as MDS referrals. However, it is important to note that the revised International Prognostic Scoring System (IPSS-R) [42] does not apply to this pathology. For CMML, a CMML-specific prognostic scoring system integrating cytogenetics has been proposed [45].

### Myeloid neoplasms with germ line predisposition

A subgroup of MDS, MDS/MPN and acute leukaemia cases are associated with a familial predisposing germ line mutation (e.g. *DDX41*, *CEBPA, GATA2, TP53* and genes involved in bone marrow failure syndromes and telomere biology disorders – see Table 7.03 WHO 2017) [1] and analysis should be performed where appropriate.

There are two constitutional chromosome abnormalities associated with predisposition to myeloid neoplasm, trisomy 21 Down syndrome and rare Robertsonian rob(15;21)(q10;q10) or der(15;21)(q10;q10). Patients with Down syndrome are at a higher risk of developing leukaemia in childhood (AML and ALL) - this also includes transient abnormal myelopoiesis (TAM). An additional copy of chromosome 21 is a rare but recurrent finding in myeloid neoplasia and therefore the finding of trisomy 21 in all cells should prompt a check regarding patient phenotype if unknown. Patients with rob(15;21) are at an increased risk (>2000-fold) of ALL with iAMP21 [46].

### Acute myeloid leukaemia (AML)

Several AML disease entities are defined by the presence of specific cytogenetic and molecular genetic abnormalities [1]. Chromosome analysis is mandatory at diagnosis for risk stratification [41]. Diagnostic workup should also include screening for molecular mutations in genes such as *NPM1*, *FLT3*, *CEPBA*, *RUNX1*, *ASXL1*, *DNMT3A*, and *TP53* and is especially important in AML with normal karyotype where these findings can define the subtype of the disease [1, 47–49]. FISH or molecular techniques may be required if a rapid result is required.

FISH analysis of interphase nuclei and metaphases to screen for *KMT2A* and *MECOM* gene rearrangements is recommended for all diagnostic AML samples if no other entity defining cytogenetic or molecular genetic abnormality is present, as these abnormalities may be cryptic and have a pronounced prognostic impact. Where karyotype has failed, or the presence of a specific abnormality is suspected clinically or morphologically but was not confirmed by cytogenetic analysis, additional FISH or molecular analysis is recommended for *PML-RARA*, *CBFB-MYH11* or *RUNX1-RUNX1T1* rearrangements. In very rare cases all three techniques may be required as the gene rearrangement may be cryptic by more than one of these approaches. For karyotype failure monosomy 5/del(5)(q31.2) and monosomy 7/del(7)(q31.2), should also be excluded. FISH may also be used where abnormalities recurrently associated with specific rearrangements are found e.g. association of inv(16) with trisomy 22. FISH testing for the cryptic t(5;11)(q35.2;p15.4)*;NUP98*-*NSD1* should be carried out in patients <5 years old with a normal karyotype due to the poor prognostic association [50–52]. Other abnormalities have been identified in paediatric AML which may require routine testing in the future: t(7;12)(q36;p13);*MNX1-ETV6* occurs mainly in infants, often accompanied by a deletion 7q [53, 54] and trisomy 19, and inv(16)(p13.3q24.3)*;CBFA2T3-GLIS2* [55, 56].

### Acute lymphoblastic leukaemia/lymphoma (ALL)

WHO 2017 and other publications [57–60] provide an overview of the non-random abnormalities found in B-lymphoblastic leukaemia/lymphoma. The most significant genetic diagnostic and prognostic factors for adult and paediatric B-ALL are: t(9;22)(q34;q11.2*);BCR-ABL1;*t(v11q23);*KMT2A* (*MLL*) rearrangements; t(12;21)(p13;q22)*;ETV6-RUNX1*; high hyperdiploidy; near haploidy; low hypodiploidy; t(1;19)(q23;p13.3);*TCF3-PBX1*; intrachromosomal amplification of chromosome 21 (iAMP21) and t(17;19)(q22;p13.3);*TCF3-HLF*. There is also a unique association between low hypodiploid ALL and *TP53* mutations which are often constitutional [61].

Screening for recurrent fusion genes by FISH/RT-PCR and chromosome banding analysis remain the gold standard methods for clinical diagnosis in BCP-ALL [57] and rapid diagnosis of clinically relevant recurrent abnormalities by FISH or RT-PCR should be undertaken. For B-ALL it is recommended that *BCR-ABL1*, *ETV6-RUNX1* and *KMT2A* gene rearrangements are routinely tested depending on the age of the patient (Table 3). This combination of probes allows the identification of other recurrent abnormalities e.g. iAMP21. Rapid diagnostic tests may be run simultaneously or sequentially (Table 3). When a *TCF3* break apart probe detects a rearrangement, it is important to distinguish between a t(17;19) and a t(1;19) as the prognosis is different. Additional FISH testing should be considered for potential high-hyperdiploidy if the karyotype is normal or is unsuccessful e.g. 4, 10, 17 and 18 [62]. For clinical trial cases it may be necessary to do further FISH analysis. A targeted FISH approach has been proposed which detects the most significant chromosomal abnormalities. Using this approach, depending on the abnormality detected, it is not necessary to carry out full banded analysis in all cases of childhood B-cell precursor ALL [57].

**Table 3:** Recommended analyses for fusion gene rearrangement testing in ALL

Patient details		Recommended	Optional
B-ALL	Infants (<1 year old)	*KMT2A*	*ETV6-RUNX1, BCR-ABL1*
	For paediatric/adolescent ALL (<25 years)	*ETV6-RUNX1, BCR-ABL1*, then *KMT2A* and *TCF3*	
	Adult	*BCR-ABL1* then *KMT2A* and *TCF3*	*ETV6-RUNX1*
T-ALL	Childhood and adult		*TLX3, TLX1, KMT2A, TAL1, LMO2* and *ABL1*

In cases where extra *RUNX1* signals are found, intrachromosomal amplification of chromosome 21 (iAMP21) is defined as 5 or more copies present in interphase nuclei (or 3 or more extra copies on as single abnormal chromosome 21 in metaphase FISH) with clustering of signals (although individual cells may display apparently distinct signals). Where extra *RUNX1* signals are not obviously clustered these are more likely to be indicative of a hyperdiploid karyotype. A hyperdiploid karyotype with more than 50 chromosomes will rarely display more than four *RUNX1* signals.

B-ALL classification includes the provisional entity B-lymphoblastic leukaemia/lymphoma, *BCR-ABL1*–like. *BCR-ABL1*–like ALL cases contain a variety of genomic alterations that activate kinase and cytokine receptor signalling pathways. These alterations can be grouped into major subclasses that include *ABL*-class fusions involving *ABL1, ABL2, CSF1R*, and *PDGFRB* that phenocopy *BCR-ABL1* and alterations of *CRLF2, JAK2* and *EPOR* that activate *JAK/STAT* signalling. This disease entity is assuming increasing importance because of its association with an adverse prognosis and response to targeted therapies. Patients with *ABL*-class fusions respond clinically to *ABL1* tyrosine-kinase inhibitors, whereas mutations activating the *JAK/STAT* pathway are amenable to treatment with *JAK* inhibitors in vitro or in preclinical models [63].

Near-haploid or low hypodiploidy is associated with a poor prognosis whereas hyperdiploidy is associated with a favourable prognosis. Near-haploid or low hypodiploid clones can ‘double-up’ and appear as hyperdiploid/near-triploid metaphases and it is therefore important to distinguish these cases from true hyperdiploid cases. Hyperdiploid clones originate from simple gain of chromosomes in a diploid cell line. Near haploid or low hypodiploid clone can easily be identified by SNP-array due to the presence of multiple whole chromosome CN-LOH.

For T-ALL, an abnormal karyotype is reported in 50–70% of cases [1]. Numerical abnormalities are less frequently observed than in B-ALL with the exception of tetraploidy, which is present in ~5% of cases. Around 35% of T-ALL have rearrangements involving the TCR loci at 7q34 (TRB) or 14q11.2 (TRA/TRD) [64, 65]. *TLX3* and *TLX1* abnormalities are observed in 25% and 5% of childhood T-ALL patients, respectively. The *NUP214-ABL1* fusion gene is found amplified as multiple (5–50) episomal copies in 6% of cases.

FISH for T-ALL is optional but could include *TLX3, TLX1, KMT2A, TAL1, LMO2* and *ABL1* rearrangements. FISH for *BCR-ABL1* (Table 3) can be used to determine the presence of the *BCR-ABL1* fusion, but also for the detection of *ABL1* amplification. Amplification of *ABL1* is indicative for the presence of *NUP214-ABL1* episomes. The presence or absence of *ABL1* amplification should be stated in the report, as this might influence treatment decisions in T-ALL [66, 67].

Many ALL cases are characterised by the presence of distinct sub-microscopic DNA copy number alterations, several of which are of clear clinical importance for risk stratification [68–70]. Array analysis is now being incorporated into clinical trials and may ultimately be introduced into routine practice. It is therefore strongly recommended that laboratories perform array analysis of all new diagnostic ALL cases.

### Chronic lymphocytic leukaemia/small lymphocytic lymphoma

Chromosomal abnormalities in chronic lymphocytic leukaemia (CLL) are detected in up to 80% of patients. The most significant genetic diagnostic and prognostic factors are deletions of 11q, 13q, 17p and trisomy 12 [71]. IWCLL guidelines [71] recommend screening for these abnormalities for pre-treatment evaluation and prognostic stratification. Suitable methodologies include FISH, array and other molecular methods. Although chromosome banding analysis has been improved with the use of oligonucleotide and IL2 it should not be considered as a standalone test as very small 13q14 deletions are frequent and cannot be identified by karyotyping. Patients showing *TP53* deletion and/or mutation are refractory to standard chemo-immunotherapy regimens [72–74]. In accordance with the European Research Initiative on CLL (ERIC) recommendations, *TP53* should be screened for deletions and point mutations prior to treatment [75]. Determination of IGHV mutational status is also mandatory [76]. Complex karyotypes with three or more abnormalities and some recurrent mutations have been reported to have a prognostic significance in CLL. Although more prospective trial data is required before it can be recommended for clinical practice [71] this testing may be included in future recommendations [77–81]. For cases of CLL with a differential diagnosis of mantle cell lymphoma (MCL) FISH to exclude a t(11;14)(q13;q32) should be performed.

### Multiple myeloma

Multiple myeloma (MM) is a neoplastic disorder characterised by a monoclonal proliferation of plasma cells in the bone marrow (or tissue) with associated acquired genetic abnormalities of clinical importance [1]. The genetic picture is frequently complex and there can be high intra-clonal variability.

The International Myeloma Working Group [82] and the European myeloma network [83] state that the minimum testing required is determination of *TP53* deletion and presence of a t(4;14)(p16;q32)*;* *FGFR3/MMSET*-IGH gene rearrangement. Testing for t(14;16)(q32;q23)*;* *MAF-*IGH gene rearrangement is also recommended [80–82]. Testing for *FGFR3-*IGH and *MAF-*IGH rearrangement can be excluded either by use of an IGH break apart probe, as a first line test, or by use of both FGFR3/IGH and MAF/IGH dual fusion probes. If an abnormal pattern is detected using an IGH break apart probe as a first line test, then further testing fort (4;14)(p16;q32)*;FGFR3/MMSET- *IGH, and t(14;16)(q32;q23)*;MAF-*IGH is required. If a single IGH dual fusion translocation probe is used as a first line test it is important to note that a normal result does not conclusively exclude the presence of any other IGH rearrangement if, for example, monosomy 14 or deletion 14q32 is present. Therefore, sufficient testing should be performed to exclude these rarer possibilities.

Other groups have included testing for 1q gain in their prognostic models [83, 84] and consequently some national guidelines have included this additional test in their recommendations [85]. 1p/1q testing is now being incorporated into clinical trials and may ultimately become an essential test. It is therefore strongly recommended that laboratories include this in their testing strategy. An extended panel may include testing for t(11;14)(q13;q32);*CCND1-*IGH, t(14;20)(q32;q12); * MAFB-*IGH, *MYC* translocations and ploidy status (if not evaluated with DNA index content) [82, 86]. When assessing hyperdiploidy by FISH the presence of any two chromosomes from a panel including 5, 9 and 15 can be considered a highly specific indicator [86]. In this rapidly changing field it is essential that laboratories keep up to date with any new recommendations for minimal essential tests. To limit the large number of FISH tests performed, MLPA or arrays can be used to assess copy number changes but FISH testing is required for IGH translocation status.

When analysing FISH, at least 100 selected plasma cells (PC) should be scored. The quality of the PC selection step should be assessed before hybridisation. In the absence of a reliable method of identifying and selecting PCs, a totally normal result must be qualified, highlighting the possibility of a false-negative result. Positive cutoff levels should be relatively conservative: 10% for fusion or break apart probes, 20% for numerical abnormalities [87].

### Other LPDs

There are no disease-specific chromosome abnormalities associated with Hairy cell leukaemia or Waldenstrom macroglobulinaemia/lymphoplasmocytic lymphoma and therefore chromosome banding analysis is not required. Mutation screening should be undertaken for these entities [1].

### B-cell lymphomas

Several recurrent cytogenetic and molecular genetic abnormalities have been described in lymphoma [1]. Genetic testing is not generally performed routinely for all lymphoma cases and is usually restricted to cases with a differential diagnosis or for prognostication purposes. Many abnormalities are not specific to a particular disease and so the result needs to be integrated with the histological reports, immunophenotype and any other genetic abnormalities. Guidelines on the diagnosis and reporting of lymphoproliferative disorders (LPD) and Lymphoma have been published that also give valuable recommendations for all laboratories/MDTs [88].

The most common recurrent chromosome abnormalities which can aid in differential diagnosis are given below. A comprehensive list can be found in WHO, 2017 or Heim & Mitelman, 2015. [1, 65]

- Mantle cell lymphoma: t(11;14);*CCND1-*IGH, and IGK/IGL and *CCND*2&3 variants.
- Follicular lymphoma: t(14;18); *BCL2-*IGH, and IGK/IGL variants, and less frequently *BCL6* rearrangements,
- Diffuse large B-cell lymphoma (DLBCL): IGH (50%), *BCL6* (30%), *BCL2*(20–30%) and *MYC* (10%) gene rearrangements.
- ALK-positive DLBCL: t(2;17)(p23;q23);*CLTC-ALK* or rarely other translocations including t(2;5)(p23;q35);*NPM1-ALK* translocation.
- Burkitt lymphoma: t(8;14)*;MYC-*IGH, and IGK/IGL variants, with no additional involvement of *BCL2* or *BCL6* or complex karyotype.
- Burkitt-like lymphoma with 11q aberration: characterised by chromosome 11q proximal gains and telomeric losses and no *MYC* rearrangement.
- Extra nodal marginal zone lymphoma of mucosa-associated lymphoid tissue: t(11;18)(q21;q21); *BIRC3-MALT1*; t(1;14)(p22;q32);*BCL10-*IGH; t(14;18)(q32;q21)*;MALT-*IGH and t(3;14)(p14.1;q32);*FOXP1-*IGH, trisomy 3 and/or trisomy 18. It should be noted that the t(14;18); *MALT1-*IGH translocation is cytogenetically identical to the t(14;18); *BCL2*-IGH translocation and that FISH is required to distinguish between them in cases with a differential diagnosis.
- T-cell prolymphocytic leukaemia: 14q11 (TRA/D) rearrangement in 80–90% T-PLL.
- Anaplastic large cell lymphoma (ALCL): *ALK* gene rearrangement, most frequent t(2;5)(p23;q35);*ALK*-*NPM1*.
- *ALK*-negative ALCL: *DUSP22-IRF4* rearrangement, most commonly a t(6;7)(p25.3;q32.3) or *TP63* rearrangements.

For prognostication, the most common referral is to exclude *MYC* rearrangement in the context of DLBCL or High grade B-cell lymphomas. High-grade B-cell lymphoma, with *MYC* and *BCL2* and/or *BCL6* translocations (so called “double-hit” or “triple-hit” lymphomas in the WHO 2008 [89]) often show complex karyotypes. For this disease entity FISH testing with IGH, * BCL2* and *BCL6* probes is essential for correct disease classification. The final classification should be combined with histology. There is no consensus or specific guidelines on when to undertake genetic testing of DLBCL. Some believe that all DLBCL should be tested for *MYC*, *BCL2* and *BCL6* rearrangements, whereas others would restrict this, to cases with a GCB phenotype and/or high-grade morphology or to cases with >40% MYC-positive cells by immunophenotype [1].

## Reporting

Reports of cytogenomic analyses should comply with ISO15189 standards and include the following information [90].

- Two unique patient identifiers (e.g. date of birth, full name—not initials);
- Sample information (type and source of sample, date of sample referral, date of report and unique sample identification);
- Referral information (reason for referral and clinical indication for test);
- Referring physician/scientist identification;
- Names of significant genes at loci involved in any established recurrent rearrangement;
- Gene names must be written following HUGO gene nomenclature (http://www.genenames.org);
- When there is fusion or rearrangement, the genes should be written as *BCR-ABL1* (i.e. use a – sign rather than a /) to distinguish the fusion product from a mixed probe kit;
- Long reports should be avoided as this detracts from the clarity of the results. Methodology and limitations of the test should not take prominence in a report as they can detract from the results and interpretation;
- Name and signature of one or two authorised persons. The signature may be generated electronically or manually;
- Pagination (i.e. page 1 of 1 or page 1 of 2);
- It is helpful to draw attention to the limitations of the analysis and any uncertainties of the result, especially when the extent of analysis has not reached the standard given in guidance documents;
- It is advisable to provide information regarding the clinical consequences of the observed genetic aberrations in the report. If a purely technical report is issued it should be made clear that the referring clinician will interpret the results and this must be clearly documented elsewhere in the patient notes;
- Where abbreviated cytogenetic results are reported for integration into a MDT-report, the information in the abbreviated MDT result must be consistent with the full cytogenetic report. The cytogenetic summary must be authorised by a suitably qualified healthcare scientist. A full version of the cytogenetic report must be sent independently to the referring health specialist.

### Analytical information

- The most recent version of ISCN should be used to report the results of chromosome banded analysis, including the number of cells;
- Single cell anomalies and heteromorphisms should not be included in the ISCN, but may be reported in the written description with qualifications;
- The FISH and array results may be given in either ISCN, a tabular format or as a summary statement in a prominent position on the report;
- The report must include the probe manufacturer, the limitations of the test (probe set), whether the analysis of the sample is restricted only to interphase cells (i.e. no metaphase analysis done), the number of normal and abnormal cells and whether the investigated material consisted of cultured or uncultured cells (only if the liquid sample has been cultured);
- Where complex results are given as a summary statement, whether or not a clinically significant abnormality was detected should be stated. The full results should be detailed elsewhere in the report;
- Cell numbers must be given for all FISH investigations in neoplastic disorders regardless of whether they are normal or abnormal, except for FFPE samples;
- It is preferable to describe the FISH results as normal or abnormal. The term ‘positive’ or ‘negative’ must not be used to describe the FISH result. Abnormal FISH results should be described as for example ‘*KMT2A* rearrangement is present in xx cells’ or ‘3 copies of *RUNX1* present in xx cells’ so that it is clear the result is not normal;
- A written description of the results should be provided including: the number of copies of any chromosome missing or extra; description of any karyotype imbalance resulting from unbalanced rearrangements; description of clinically relevant structural abnormalities, including genes in the rearrangement. This is particularly important if the results are given in ISCN which may not be interpretable by the reader of the report;
- FISH manufacturer and array platform used should be included in the report;
- The genome build, if required, should be included either in the ISCN or appear elsewhere in the report for abnormal results;
- Limitations of the test(s) used should be given.

### Interpretation

- Relationship of any abnormalities found to the referral reason, or other possible disease association where appropriate (differential diagnosis, abnormality not related to referral reason);
- Although mosaic trisomy 8 can be constitutional, it is not considered necessary to attempt to exclude this in the majority of cases where an extra chromosome 8 is found as the sole abnormality in a myeloid disorder. When reporting –Y or +15 it should be made clear that these changes can be found in elderly patients with no haematological neoplasm [91–93];
- The WHO 2017 nomenclature should be used in relation to the disease category, where appropriate [1];
- The generic term “malignancy” should not be used in the context of a clone of unknown significance. The term ‘chromosomally aberrant clone’ is recommended instead;
- Prognosis if robust data from multiple publications/ international trials/trial protocols exists: e.g. evidence from large randomised control trials of patients undergoing similar relevant treatment or meta-analysis/systematic review of multiple studies. Multiple concordant studies can be used and should be referenced, Small and isolated studies should not be used to derive prognosis;
- Recommendations for other investigations (e.g. FISH) to clarify significance of the results.

### Reporting times

The guidance in Table 4 (below) is for maximum reporting times and it is expected that the majority of referrals will be reported well within these times. The laboratory should have contingencies for providing rapid reporting of some results.

**Table 4:** Recommended reporting times

Urgent referrals (e.g. acute leukaemia):	95% should be reported within 10 calendar days. A diagnostic FISH result is adequate in this category, with confirmatory chromosome banding analysis treated as for routine referrals.
Rapid test by FISH/PCR (e.g. *RARA* rearrangements)	95% reported in 3 working days. A result should be given in <24 h.
Routine referral (e.g. follow-up):	90% should be reported within 21 calendar days.
